# An Ethanolic Extract of *Lindera obtusiloba* Stems, YJP-14, Improves Endothelial Dysfunction, Metabolic Parameters and Physical Performance in Diabetic db/db Mice

**DOI:** 10.1371/journal.pone.0065227

**Published:** 2013-06-03

**Authors:** Jung-Ok Lee, Cyril Auger, Dong Hyun Park, Moonkyu Kang, Min-Ho Oak, Kyoung Rak Kim, Valérie B. Schini-Kerth

**Affiliations:** 1 UMR CNRS 7213, Laboratoire de Biophotonique et Pharmacologie, Faculté de Pharmacie, Université de Strasbourg, Illkirch, France; 2 Research and Development Center, Hanwha Pharma. Co., Ltd., Chuncheon, Republic of Korea; 3 Research Center, YangJi Chemicals, Suwon, Republic of Korea; 4 College of Pharmacy, Mokpo National University, Muan, Jeonam, Republic of Korea; The Chinese University of Hong Kong, Hong Kong

## Abstract

*Lindera obtusiloba* is a medicinal herb traditionally used in Asia for improvement of blood circulation, treatment of inflammation, and prevention of liver damage. A previous study has shown that an ethanolic extract of *Lindera obtusiloba* stems (LOE) has vasoprotective and antihypertensive effects. The possibility that *Lindera obtusiloba* improves endothelial function and metabolic parameters in type 2 diabetes mellitus (T2DM) remains to be examined. Therefore, the aim of the present study was to determine the potential of LOE to prevent the development of an endothelial dysfunction, and improve metabolic parameters including hyperglycemia, albuminuria and physical exercise capacity in db/db mice, an experimental model of T2DM. The effect of LOE (100 mg/kg/day by gavage for 8 weeks) on these parameters was compared to that of an oral antidiabetic drug, pioglitazone (30 mg/kg/day by gavage). Reduced blood glucose level, body weight and albumin-creatinine ratio were observed in the group receiving LOE compared to the control db/db group. The LOE treatment improved endothelium-dependent relaxations, abolished endothelium-dependent contractions to acetylcholine in the aorta, and normalized the increased vascular oxidative stress and expression of NADPH oxidase, cyclooxygenases, angiotensin II, angiotensin type 1 receptors and peroxynitrite and the decreased expression of endothelial NO synthase in db/db mice. The angiotensin-converting enzyme (ACE) activity was reduced in the LOE group compared to that in the control db/db group. LOE also inhibited the activity of purified ACE, COX-1 and COX-2 in a dose-dependent manner. In addition, LOE improved physical exercise capacity. Thus, the present findings indicate that LOE has a beneficial effect on the vascular system in db/db mice by improving endothelium-dependent relaxations and vascular oxidative stress most likely by normalizing the angiotensin system, and also on metabolic parameters, and these effects are associated with an enhanced physical exercise capacity.

## Introduction

The prevalence of type 2 diabetes mellitus (T2DM), which is characterized by insulin resistance sometimes associated with relative insulin deficiency, is continuously increasing in westernized societies due to the aging population, the increased prevalence of obesity and sedentary lifestyles [1], [2], [3]. T2DM is a metabolic disorder of multiple etiologies characterized by chronic hyperglycemia, which results in the development of diabetes-related complications such as cardiovascular diseases, nephropathy, neuropathy and retinopathy [4], [5], [6]. It has been estimated that more than 80% of patients with T2DM have major cardiovascular diseases such as coronary artery diseases, heart failure and peripheral artery diseases [7], [8], [9].

An endothelial dysfunction characterized by blunted endothelium-dependent vasorelaxation is observed early in the development of diabetes mellitus and has been suggested to be a key event in the initiation and development of both macro-vascular and micro-vascular complications in T2DM [10], [11], [12]. Indeed, reduced flow-mediated dilation of the brachial artery has been observed in clinical studies [13], [14], and blunted endothelium-dependent relaxations of isolated arteries in several experimental models of T2DM such as the leptin receptor deficient db/db mice, Goto-Kakizaki rats, Otsuka Long-Evans Tokushima fatty rats, and Zucker diabetic fatty rats [12], [15]. The characterization of the blunted endothelium-dependent relaxations in T2DM has indicated the involvement of reduced nitric oxide (NO) and endothelium-dependent hyperpolarization (EDH) components, two major endothelium-derived vasorelaxing mechanisms [16], [17]. Moreover, the endothelial dysfunction is related to increased oxidative stress in the arterial wall involving increased formation of superoxide anion and hydrogen peroxide, predominantly due to an up-regulation of NADPH oxidase throughout the arterial wall, and possibly also to an uncoupling of endothelial NO synthase (eNOS) [18]. Reactive oxygen species (ROS) such as superoxide anions may reduce the NO bioavailability by chemically reacting with NO to generate peroxynitrite, but also by reducing the bioavailability of tetrahydrobiopterin (BH_4_), an essential cofactor of eNOS [19], [20]. In addition, oxidative stress has also been associated with blunted EDH-mediated relaxations, at least in part, by reducing the expression of both small and intermediate conductance calcium-dependent potassium channels (SK_Ca_ and IK_Ca_, respectively) [21]. The endothelial dysfunction in T2DM has also been associated with the induction of endothelium-dependent contractile responses involving cyclooxygenase-derived metabolites of arachidonic acid (AA) acting on TP receptors to contract the vascular smooth muscle [22].

Several lines of evidence suggest that the angiotensin system contributes to the impaired endothelial function in T2DM. Indeed, angiotensin-converting enzyme (ACE) inhibitors and angiotensin II (Ang II) receptor type I blockers prevented endothelial dysfunction in diabetic animals and humans [23], [24]. Moreover, Ang II is a potent inducer of endothelial dysfunction and NADPH oxidase-derived vascular oxidative stress [23], [25].

Stems of *Lindera obtusiloba* have been used to treat bruises, blood stasis, and swelling in the Korean traditional medicine [26]. Moreover, our previous study has indicated that an ethanolic extract of *Lindera obtusiloba* stems (LOE) at a dose of 100 mg/kg/day prevented endothelial dysfunction and hypertension induced by the chronic infusion of Ang II to rats, in part, by normalizing the NADPH oxidase-dependent vascular oxidative stress [27]. Therefore, the aim of the present study was to determine whether LOE prevents endothelial dysfunction in an experimental model of T2DM, the db/db mice, through inhibition of oxidative stress and the angiotensin system.

## Results

### LOE Improves Clinical Parameters in db/db Mice

As expected, the weight gain increased to a greater extent in the db/db group compared to the db/+ group from week 6 until week 14 (Table 1). The LOE treatment significantly reduced the weight gain in the db/db group by about 18.6±7% whereas the weight gain was more pronounced in the pioglitazone group (the increase amounted to about 25.6±4.5%, Table 1). The intake of food, which was determined every week, was significantly greater in the db/db group compared to that in the db/+ group, and this effect was affected neither by the pioglitazone treatment nor the LOE treatment (Table 1). At week 14, the liver weight in the db/db group was significantly greater than that of the db/+ group (Table 1). The LOE treatment reduced slightly but not significantly the liver weight by 15.4±3.8% whereas the pioglitazone treatment increased it significantly by 46.2±7.8% (Table 1). The heart weight was similar in the db/+ group and the db/db group, and it was not affected by the LOE treatment but significantly increased by the pioglitazone treatment by about 25±9.9% (Table 1). The kidney weight was similar in the 4 groups tested (data not shown).

**Table 1:** Effect of LOE and pioglitazone on body weight, organ weight and food intake in db/db mice.

	db/+	db/db	db/db+Pio	db/db+LOE
Body weight (6 weeks), g	24.0±0.3	30.5±0.7*	29.6±0.7*	30.5±0.6*
Body weight (14 weeks), g	27.3±0.4	51.5±1.1*	57.7±1.1* ^,^ #	47.0±1.7* ^,^ #
Liver weight, g	1.4±0.03	2.6±0.1*	3.8±0.2* ^,^ #	2.2±0.1*
Heart weight, mg	141±3	142±5	178±14*	132±6
Food intake (14 weeks),g/24 h	4.4±0.5	6.6±0.5*	5.8±0.3*	5.8±0.4*

Values are shown as means ± S.E.M. (n = 13–14).

* *P*<0.05 indicates a significant difference versus the db/+ group,

# *P*<0.05 indicates a significant difference between db/db group and db/db+Pio group or db/db+LOE group.

### LOE Improves Metabolic Parameters in db/db Mice

**Figure 1:**
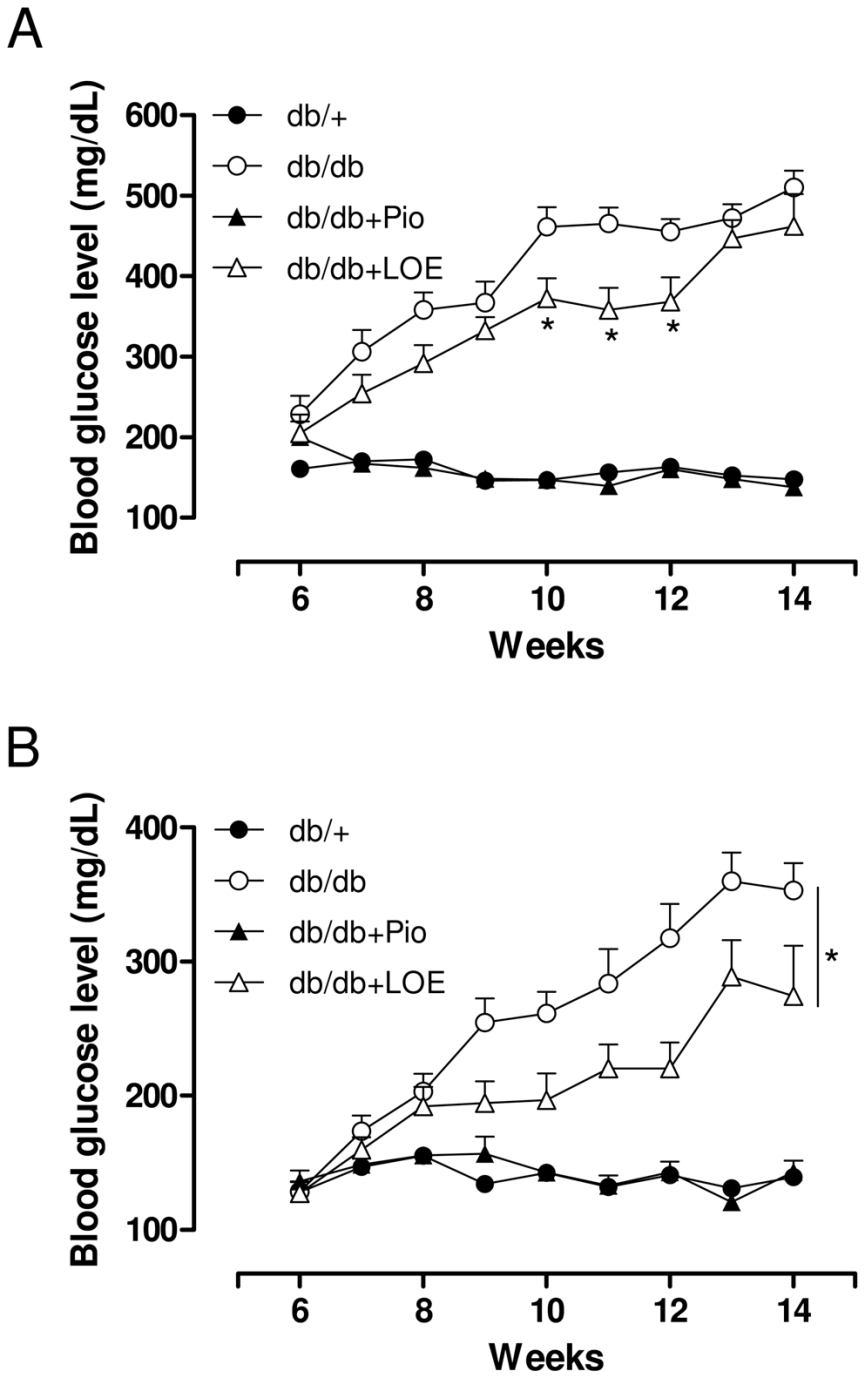
LOE and pioglitazone treatments improve blood glucose levels in db/db mice. (A) Non-fasting and (B) fasting blood glucose levels from week 6 until week 14. All groups were fasted for 4 h before blood samples were taken. Values are shown as means ± S.E.M. (n = 13–14). *P<0.05 indicates a significant difference versus db/db group.

Both non-fasting and fasting blood glucose levels increased markedly from week 6 until week 14 in the db/db group whereas they remained unchanged in the db/+ group (Fig. 1 A and B). LOE treatment significantly reduced non-fasting blood glucose levels from week 10 until week 12, and also of fasting blood glucose levels from week 9 until week 14 in the db/db group (Fig. 1 A and B). In contrast, pioglitazone abolished the increase in both the non-fasting and fasting blood glucose levels (Fig. 1 A and B).

The determination of metabolic parameters in plasma indicated that GOT and GPT levels were similar in db/+ mice and db/db mice, and that the LOE treatment did not affect both parameters whereas the GPT level but not the GOT level was significantly increased by the pioglitazone treatment (the increase amounted to 480±89.2% compared to that of the db/db group, Table 2). Analysis of the lipid profile showed that the CHO, TG and HDL levels were significantly increased in db/db mice compared to those in db/+ mice whereas the LDL levels were similar (Table 2). The LOE treatment did not significantly affect the CHO, HDL and LDL levels but significantly reduced that of TG by 65.5±9.1% in db/db mice (Table 2). The pioglitazone treatment significantly increased the CHO level by 69.1±18.3%, affected minimally the LDL and the HDL level, and decreased markedly that of TG by 114.5±7.5% in db/db mice (Table 2).

**Table 2:** Effect of LOE and pioglitazone on metabolic parameters in plasma of db/db mice.

	db/+	db/db	db/db+Pio	db/db+LOE
GOT, mg/dl	145.0±13.1	160.2±17.4	205.1±14.6*	197.1±15.4
GPT, mg/dl	37.9±9.8	78.8±9.8	275.1±36.5* ^,^ #	77.1±12.7
CHO, mg/dl	56.0±3.1	89.4±3.5*	112.5±6.1* ^,^ #	82.0±4.8*
TG, mg/dl	58.0±7.2	191.4±26.0*	38.7±10.0* ^,^ #	104.0±12.2* ^,^ #
HDL, mg/dl	30.5±2.5	49.6±1.4*	56.5±2.6*	46.3±3.4*
LDL, mg/dl	4.4±0.6	5.3±0.6	7.0±0.7*	4.9±0.4

Values are shown as means ± S.E.M. (n = 13–14).

* *P*<0.05 indicates a significant difference versus the db/+ group,

# *P*<0.05 indicates a significant difference between db/db group and db/db+Pio group or db/db+LOE group.

### LOE Prevents Albuminuria in db/db Mice

As expected, the db/db group had markedly increased 24-h urinary volume compared to that of the db/+ group, this effect was not affected by the LOE treatment but abolished by the pioglitazone treatment (Table 3). The albumin-creatinine ratio (ACR, an indicator of renal function) was markedly increased by about 335.2±6.1% in the db/db group compared to the db/+ group, and this effect was significantly reduced by the LOE treatment by about 36.2±0.5% but not by the pioglitazone treatment (Table 3).

**Table 3:** Effect of LOE and pioglitazone on urinary volume and renal function in db/db mice.

	db/+	db/db	db/db+Pio	db/db+LOE
Urinary volume, ml	0.84±0.13	8.31±1.66*	0.79±0.13#	8.45±1.25*
ACR, mg/g	22.44±0.60	75.23±1.38*	69.90±0.58*	48.00±0.46* ^,^ #

ACR: albumin-creatinine ratio. Values are shown as means ± S.E.M. (n = 13–14).

* *P*<0.05 indicates a significant difference versus the db/+ group,

# *P*<0.05 indicates a significant difference between db/db group and db/db+Pio group or db/db+LOE group.

### LOE Improves Endothelium-dependent Relaxations and Prevents Endothelium-dependent Contractions in db/db Mice

**Figure 2:**
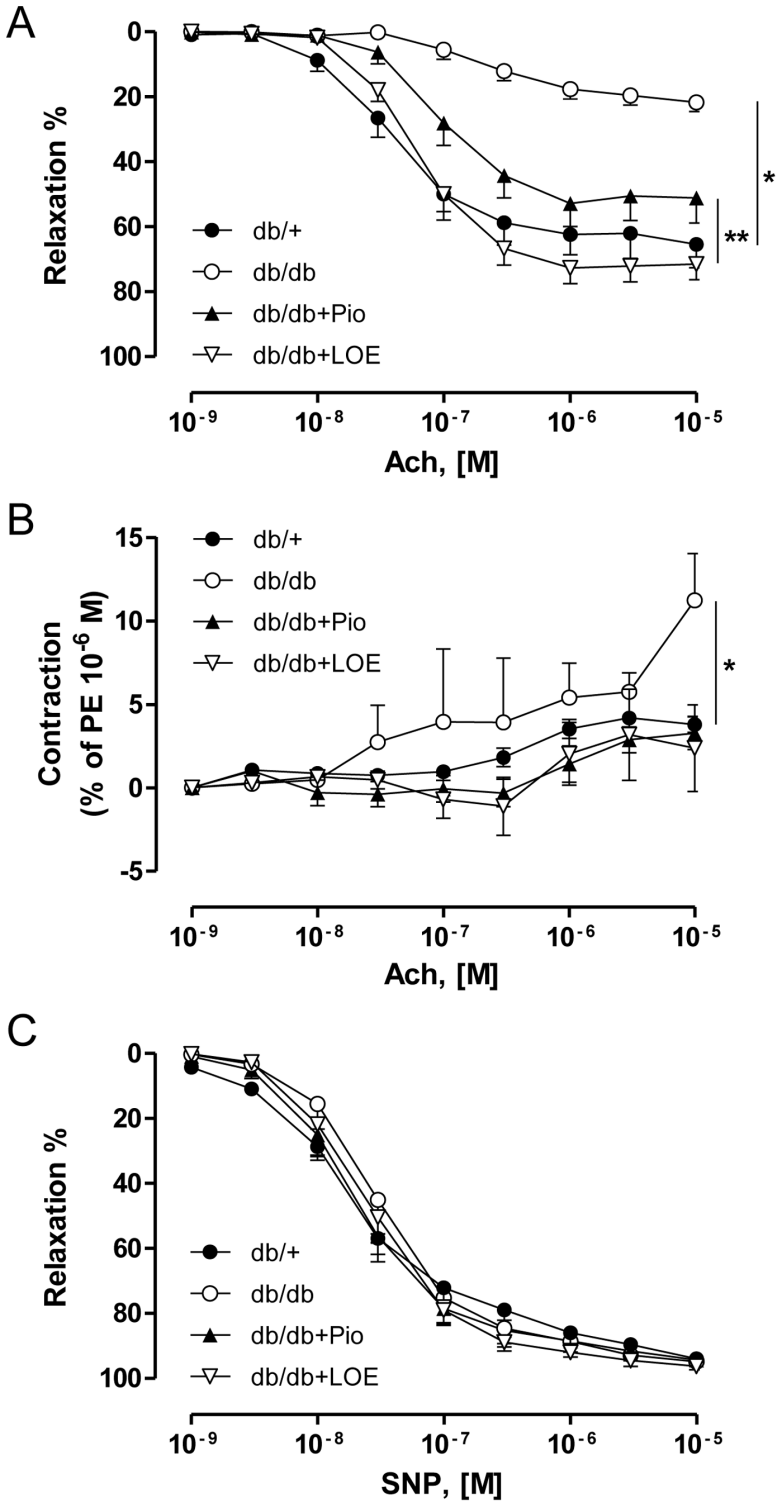
LOE and pioglitazone treatments improve endothelium-dependent relaxations and reduce endothelium-dependent contractile responses to acetylcholine in aortic rings of db/db mice. (A) Concentration-relaxation curves to Ach in aortic rings with endothelium, (B) concentration-contraction curves to Ach in the presence of N^w^-nitro L-arginine (an inhibitor of eNOS) in rings with endothelium, and (C) concentration-relaxation curves to sodium nitroprusside (a NO donor) in rings with endothelium in the presence of N^w^-nitro L-arginine and indomethacin to avoid the formation of endothelium-derived vasoactive NO and prostanoids, respectively. Values are shown as means ± S.E.M. (n = 5–6). *P<0.05 indicates a significant difference between db/+ group versus db/db group, db/db+Pio group or db/db+LOE group, and † db/db+Pio group versus db/db+LOE group.pone.0065227.g003.tif

**Figure 3:**
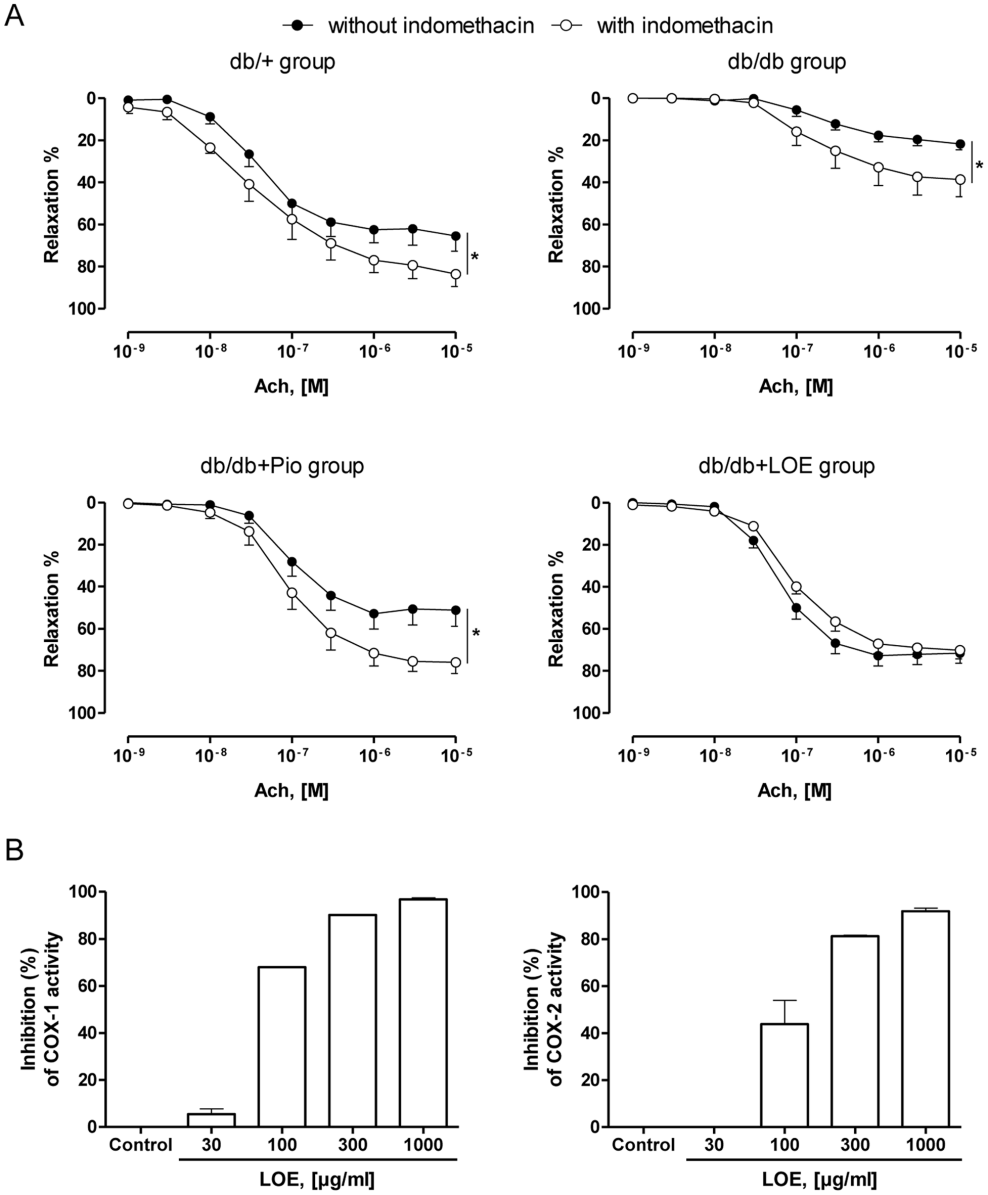
LOE inhibits the activity of cyclooxygenases. (A) Cyclooxygenase-derived vasoactive prostanoids blunt endothelium-dependent relaxations to Ach in aortic rings of the db/+, db/db and db/db+Pio groups but not in the db/db+LOE group. Concentration-dependent relaxations induced by Ach in aortic rings with endothelium in the absence and presence of indomethacin (an inhibitor of cyclooxygenases). Values are shown as means ± S.E.M. (n = 5–7). *P<0.05 indicates a significant difference versus the respective control. (B) LOE inhibits COX-1 and COX-2 activity *in vitro* in a concentration-dependent manner. Values are shown as means ± S.D. (duplicate determinations).

Ach caused concentration-dependent relaxations in aortic rings, which reached about 65.5% at 10 µM in the db/+ group but only about 21.7% in the db/db group (Fig. 2A). Both the pioglitazone and the LOE treatment improved markedly endothelium-dependent relaxations to Ach in the db/db group (Fig. 2A; maximal relaxations were 51.1% and 72.7%, respectively). In the presence of *N* ^ω^-nitro-L-arginine to prevent the endothelial formation of NO, Ach at 10 µM caused small but significant contractions in aortic rings with endothelium in the db/db group but not in the db/+ group, the db/db+Pio group and the db/db+LOE group (Fig. 2B). The NO donor SNP caused similar relaxations in aortic rings in all four groups (Fig. 2C). In addition, indomethacin, an inhibitor of cyclooxygenases, slightly but significantly improved relaxations to Ach in the db/+, the db/db and the db/db+Pio groups whereas no such effect was observed in the db/db+LOE group (Fig. 3A). The possibility that LOE is able to inhibit COX-1 and COX-2 activity was assessed using purified enzymes. As shown in Figure 3B, LOE inhibited in a concentration-dependent manner purified COX-1 and COX-2 with IC_50_ of 85.4±1.38 and 104.7±1.12 µg/ml, respectively.

### LOE Decreases Vascular Oxidative Stress and the Up-regulation of NADPH Oxidase Subunits and Cyclooxygenases in db/db Mice

**Figure 4:**
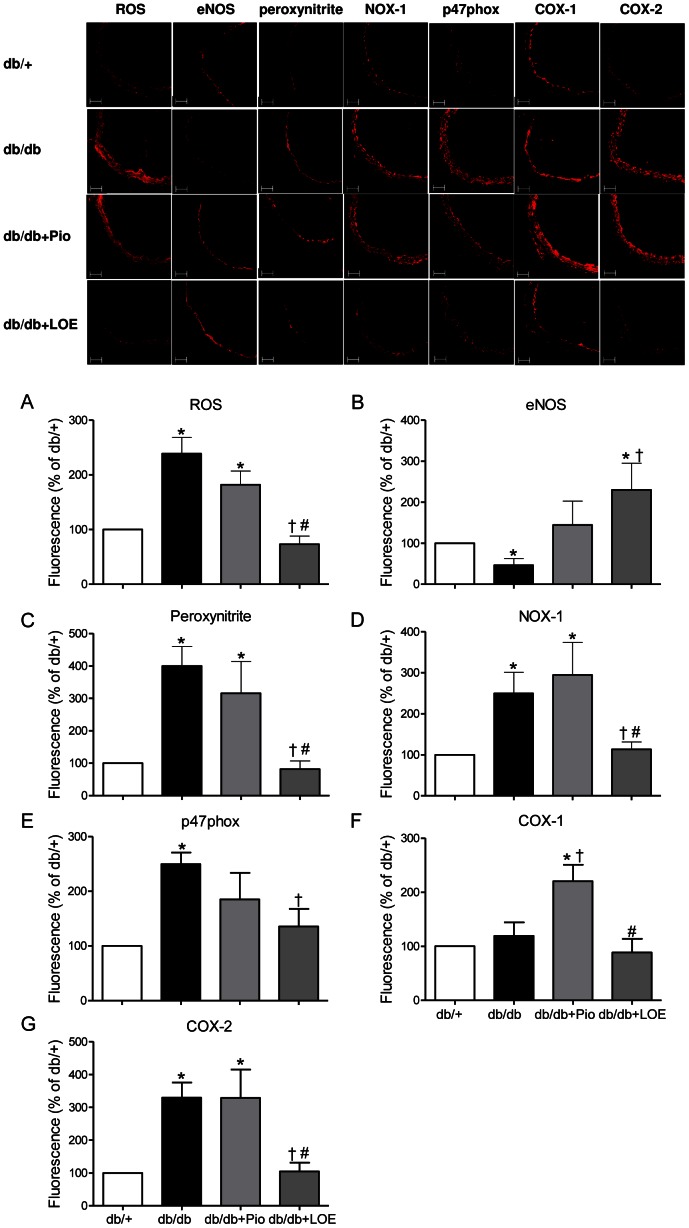
Effect of LOE and pioglitazone treatments on vascular oxidative stress, and the expression of eNOS, NADPH oxidase subunits (NOX-1 and p47phox) and cyclooxygenases (COX-1 and COX-2) in aortic sections of db/db mice. The formation of reactive oxygen species (ROS) was determined using the redox-sensitive probe dihydroethidine, and the expression of target proteins by immunofluorescent staining. Top: representative immunofluorescent staining; bottom: corresponding cumulative data. The lumen is on the right side of each image, and the scale bar corresponds to 50 µm. Values are shown as means ± S.E.M. (n = 6–7). *P<0.05 indicates a significant difference between db/+ group versus db/db group, db/db+Pio group or db/db+LOE group, ^†^db/db group versus db/db+Pio group or db/db+LOE group, and ^#^db/db+Pio group versus db/db+LOE group.

A marked increase of the fluorescence ethidium signal was observed throughout the aortic wall in the db/db group compared to the db/+ group (the increase amounted to 238.7±29.7%) indicating an increased level of oxidative stress (Fig. 4A). This effect was associated with an enhanced expression of the NADPH oxidase subunits NOX-1 (249.7±51.6%) and p47phox (249.9±21.2%), and peroxynitrite as assessed by nitrotyrosine fluorescence (399.4±60.3%, Fig. 4C, D and E). In the db/db group, an increased vascular expression level of COX-2 (329.5±46.0%, Fig. 4G) was observed whereas that of COX-1 was similar (Fig. 4F) and that of eNOS was reduced compared to the db/+ group (46.4±15.9%, Fig. 4B). The LOE treatment significantly decreased the formation of ROS and peroxynitrite, and the expression of NOX-1, p47phox, and COX-2, up-regulated that of eNOS, and did not affect that of COX-1 in the db/db group (Fig. 4). Compared to db/db+Pio group, the LOE treatment significantly decreased the formation of ROS and peroxynitrite, and the expression of NOX-1, COX-1, and COX-2, and it up-regulated that of eNOS (Fig. 4). In contrast, the pioglitazone treatment did not affect the increased level of oxidative stress, peroxynitrite, NOX-1, p47phox and COX-2 in aortic sections of the db/db group but it markedly increased the expression level of COX-1, and slightly but not significantly that of eNOS (Fig. 4).

### LOE Reduces the Vascular Angiotensin System in db/db Mice

**Figure 5:**
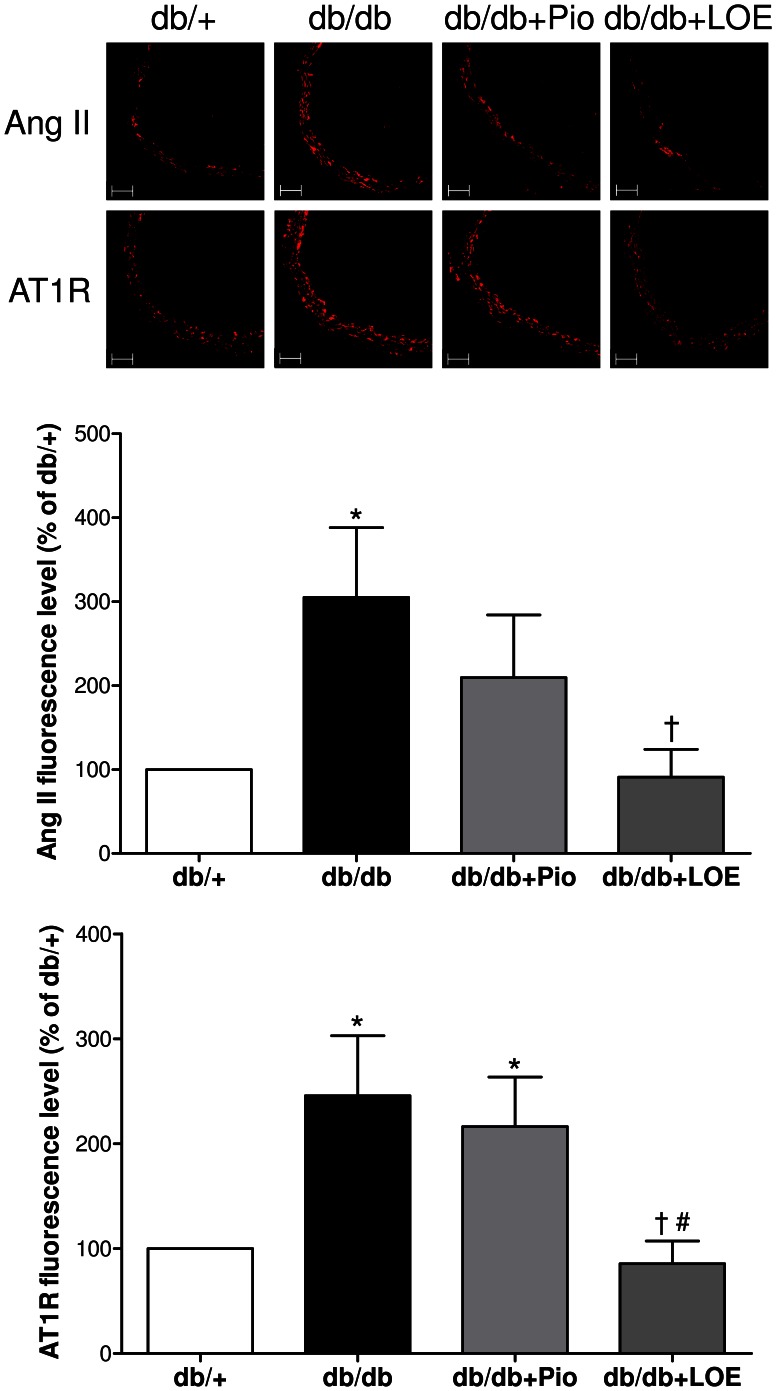
Effect of LOE and pioglitazone treatments on the expression of angiotensin II (Ang II) and angiotensin II type 1 receptors (AT1R) in aortic sections of db/db mice. The expression of Ang II and AT1R was assessed by immunofluorescent staining. Top: representative immunofluorescent staining; bottom: corresponding cumulative data. The lumen is on the right side of each image, and the scale bar corresponds to 50 µm. Values are shown as means ± S.E.M. (n = 6–7). *P<0.05 indicates a significant difference between db/+ group versus db/db group, db/db+Pio group or db/db+LOE group, ^†^db/db group versus db/db+Pio group or db/db+LOE group, and ^#^db/db+Pio group versus db/db+LOE group.

**Figure 6:**
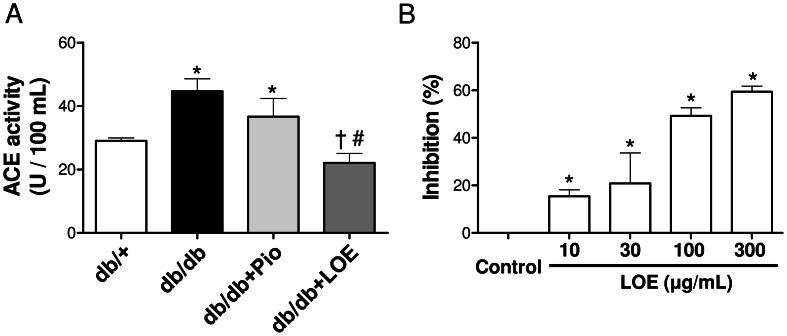
LOE reduces plasma angiotensin converting enzyme (ACE) activity in db/db mice (A), and inhibits the activity of purified ACE (B). Values are shown as means ± S.E.M. n = 6–7 (A) and 3 (B). *P<0.05 indicates a significant difference between db/+ group versus db/db group, db/db+Pio group or db/db+LOE group, ^†^db/db group versus db/db+Pio group or db/db+LOE group, and ^#^db/db+Pio group versus db/db+LOE group.

Since previous studies have shown that LOE prevented Ang II-induced hypertension and endothelial dysfunction in rats [27], and that increased levels of Ang II and its major receptor, Ang II type 1 receptor (AT1R), are observed in aortic sections of db/db mice [28], the possibility that LOE affects the local angiotensin system was assessed. The expression level of Ang II and AT1R in aortic sections was significantly increased to about 305.0±83.2% and 245.7±57.2%, respectively, in the db/db group compared to the db/+ group (Fig. 5A and 5B). The pioglitazone treatment did not affect Ang II and AT1R levels whereas the LOE treatment markedly reduced those to 90.9±33.0% and 85.6±21.6%, respectively (Fig. 5A and 5B). The possibility that the LOE treatment affects the ACE activity was assessed in the plasma. An increased ACE activity was observed in the db/db group compared to the db/+ group (44.7±3.9 and 29±0.9 U/100 ml, respectively), and the LOE treatment abolished the ACE activity (22±3.0 U/100 ml) whereas the pioglitazone treatment (36.7±5.8 U/100 ml) was without effect (Fig. 6A). In addition, LOE inhibited the enzymatic activity of a purified preparation of porcine ACE in a concentration-dependent with an IC_50_ of 123.3 µg/ml (Fig. 6B).

### LOE Improves the Physical Performance of db/db Mice

**Figure 7:**
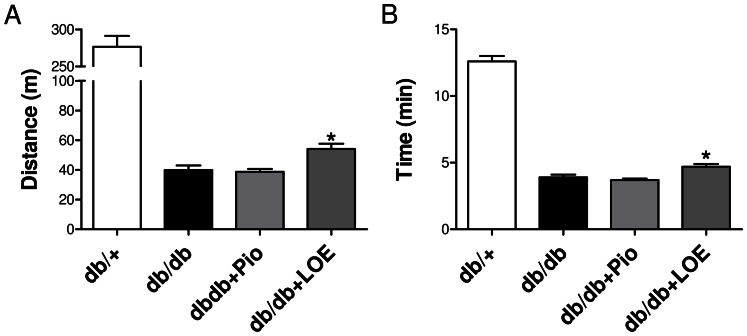
LOE but not pioglitazone improves slightly but significantly the physical exercise capacity of db/db mice. The physical performance was assessed using a treadmill. (A) Time to exhaustion, (B) maximal distance run before exhaustion. Values are shown as means ± S.E.M. (n = 13–14). *P<0.05 indicates a significant difference versus db/db group.

A severely impaired physical exercise performance is often observed in experimental models of obesity including the db/db mice [29]. Therefore, experiments were performed to determine whether the LOE treatment improves the physical exercise capacity as assessed by the time and distance to exhaustion using a treadmill. The db/db group showed a markedly impaired physical exercise capacity (the time to exhaustion was reduced by 69.0% and the distance by 85.6% compared to the db/+ group, Fig. 7A and 7B). The impaired responses were not affected by the pioglitazone treatment but slightly but significantly improved by the LOE treatment by about 6.3% and 5.1% compared to the db/db group, respectively (Fig. 7A and 7B).

## Discussion

The major findings of the present study indicate that chronic intake of LOE by db/db mice improved the endothelial dysfunction and the excessive vascular oxidative stress most likely by normalizing the angiotensin system, and also several metabolic parameters including hyperglycemia; all these effects were associated with an improved physical exercise performance.

In T2DM, a disorder characterized by hyperglycemia and insulin resistance, the development of an endothelial dysfunction is often an early marker, which has gained interest since it is thought to promote the development of diabetic microvascular and macrovascular complications such as cardiovascular diseases and retinopathy [30]. Thus, the search for novel treatments capable to improve, besides hyperglycemia, the endothelial function is one of the most promising goals to prevent diabetic vascular complications [31]. Previous studies have indicated that several polyphenol-rich natural products are able to induce endothelium-dependent relaxations by increasing the endothelial formation of major vasoprotective factors such as NO and EDH, and also to prevent and/or improve an established endothelial dysfunction in several types of cardiovascular diseases such as hypertension [32], [33], [34], [35], [36]. In addition, our previous investigations have indicated that LOE is a potent inducer of NO-mediated relaxations in aortic rings and stimulator of the PI3-kinase/Akt-dependent phosphorylation of eNOS at the activator site Ser 1177 and, also, that LOE prevented the angiotensin II-induced hypertension and endothelial dysfunction in rats [27]. The present findings indicate that chronic administration of LOE to db/db mice, an experimental model of T2DM, fully restored the severely blunted endothelium-dependent relaxations to Ach in aortic rings as well as improved partially hyperglycemia. In addition, since the LOE treatment affected minimally relaxations induced by the NO donor, SNP, and the endothelium-dependent relaxation to Ach in aortic rings is abolished by *N* ^ω^-nitro-L-arginine, an inhibitor of eNOS (data not shown), the beneficial effect of the LOE treatment can be attributed to an improved endothelial NO-mediated response rather than an improved cyclic GMP-dependent relaxing pathway in the vascular smooth muscle. Interestingly, the oral anti-diabetic drug, pioglitazone, normalized blood glucose levels in db/db mice and it also improved the endothelial dysfunction. In addition, the endothelial dysfunction in aortic rings of db/db mice involves also the induction of small but significant endothelium-dependent contractile responses to Ach, which were prevented by both the LOE and the pioglitazone treatments. Moreover, vasoconstrictor prostanoids also seem to blunt the Ach-induced relaxations in both the db/+ and the db/db group since these responses were potentiated by the cyclooxygense inhibitor, indomethacin. Although a similar potentiating effect was observed in the pioglitazone-treated group, no such effect was observed in the LOE-treated group suggesting that LOE might inhibit cyclooxygenases. Indeed, the *in vitro* determination of cyclooxygenase activity indicated that LOE inhibited the enzymatic activity of both COX-1 and COX-2 in a similar manner.

Numerous studies have indicated that diabetes mellitus and hyperglycemia are associated with an excessive vascular formation of ROS, which largely accounts for the impaired endothelial NO component due to the fact that NO can chemically react with superoxide anions leading to the subsequent formation of peroxynitrite but possibly also by oxidizing BH_4_, an essential cofactor of eNOS leading to eNOS uncoupling [37]. Indeed, vitamin C has been shown to correct endothelial dysfunction in patients with either type 1 diabetes mellitus (T1DM) or T2DM [38], [39], [40], and also in several animal models of diabetes mellitus such as a streptozotocin-induced model [41], [42]. Moreover, oral glucose loading in healthy subjects without diabetes mellitus caused an acute and transient decrease of flow-mediated dilation, which was prevented by the vitamins C and E [43]. Several vascular sources of ROS have been involved in the diabetes-associated vascular oxidative stress including the NADPH oxidase, uncoupled eNOS, xanthine oxidase and mitochondrial dysfunction [18], [44]. Consistent with these previous findings, an increased level of vascular oxidative stress was observed in db/db mice as indicated by the marked ethidium staining throughout the arterial wall, and the increased peroxynitrite, which was predominantly observed at the luminal surface in the aorta of db/db mice. A major role for the NADPH oxidase among several sources of ROS in the arterial well is supported by the fact that an increased expression of both NADPH oxidase subunits NOX-1 and p47phox is observed throughout the arterial wall of db/db mice. The beneficial effect of the LOE treatment on the endothelium-dependent NO-mediated relaxation most likely involves its ability to normalize vascular oxidative stress, in part, by normalizing the expression of NADPH oxidase but also by restoring the blunted eNOS expression in db/db mice. In several previous studies, polyphenols have been shown to improve in part vascular oxidative stress due to their direct antioxidant properties, their ability to inhibit NADPH oxidase activity [45], and possibly also to the induction of changes in the expression pattern of endogenous pro-oxidant and anti-oxidant enzymes in the arterial wall [46], [47], [48]. An improved vascular oxidative stress and endothelium-dependent NO-mediated relaxation in response to polyphenol-rich products has also been observed in several experimental models of diabetes mellitus and vascular diseases including hypertension and ageing [27], [32], [49], [50]. Such a combination of effects most likely accounts for the effectiveness of the LOE treatment to restore a normal endothelial function and vascular oxidative stress level despite the persistent hyperglycemia in db/db mice. In addition, the endothelial dysfunction in db/db mice appears to involve, besides oxidative stress, also several additional key mechanisms since normalization of the hyperglycemia by the pioglitazone treatment improved endothelial dysfunction despite not affecting vascular oxidative stress.

Numerous studies have shown that in addition to hyperglycemia, dyslipidemia and chronic inflammation of the arterial wall are often observed in T2DM and are thought to contribute to its progression and vascular complications [31], [51]. Indeed, an elevation of the triglyceride-rich lipoproteins level is likely to promote the formation of non-esterified fatty acids (NEFA) and the up-regulation of NADPH oxidase via activation of lectin-type oxidized LDL receptor 1, and an elevation of the low-density lipoprotein (LDL) level, which will result in the accumulation of lipids into macrophages and, hence, plaque growth [31]. Moreover, numerous studies have shown that both hypertriglyceridemia and hypercholesterolemia trigger the development of an endothelial dysfunction associated with vascular oxidative stress [52], [53], [54]. In the present study, an increased TG level was observed in the db/db group compared to the db/+ group, which was significantly reduced by both the LOE and the pioglitazone treatments. In addition, although the db/db group had similar LDL levels as the db/+ group, the pioglitazone treatment induced a significant increase in LDL levels whereas the LOE treatment was without effect. The possibility that the pioglitazone-induced upregulation of LDL levels might counteract the beneficial effect of the glitazone on the endothelial function remains to be studied.

The fact that both ACE inhibitors and AT1R blockers prevented the endothelial dysfunction in diabetic animals and humans indicates a determinant role of the angiotensin system [23], [24]. Moreover, an age-related increase in blood pressure has been observed in db/db mice, which was reduced by an AT1 receptor antagonist [55]. Therefore, the possibility that the LOE and/or the pioglitazone treatments improve the angiotensin system was evaluated by determining the expression level of several members of the angiotensin system including Ang II, AT1R, and ACE activity in the arterial wall. Significantly increased expression levels of both Ang II and AT1R were observed throughout the arterial wall in the db/db group possibly reflecting the higher ACE activity. The LOE treatment normalized all these responses whereas the pioglitazone treatment was without effect. In addition, LOE inhibited strongly partially purified ACE with an IC_50_ of 123.3 µg/ml. Altogether, these findings indicate that the inhibitory effect of LOE on the angiotensin system is likely to contribute to its beneficial effect on the endothelial dysfunction and vascular oxidative stress.

In order to better evaluate the potential beneficial effect of the LOE treatment on T2DM, changes of body weight, food intake, tissue weight, renal function, urinary volume and physical exercise capacity were also determined throughout this study. As expected, a pronounced increase in body weight was observed in the db/db mice compared to the db/+ mice; this effect was partially reduced by the LOE treatment possibly due to its ability to inhibit adipogenesis via a sustained Wnt signaling [56]. In contrast, the pioglitazone treatment increased body weight in the db/db group; such an effect is also often observed in treated diabetic animals and humans [57], [58]. In addition, db/db mice had an increased liver weight reflecting most likely a fatty liver due to obesity whereas the heart weight was normal. The LOE treatment did affect neither the liver weight nor the heart weight whereas both were significantly increased by the pioglitazone treatment.

The development of diabetes mellitus is often associated with the induction of renal dysfunction. Indeed, diabetic nephropathy is a major end-stage renal disease in USA, Europe and Japan [59]. Clinically, diabetic nephropathy can be diagnosed by albuminuria, which can be identified by ACR [60]. Therefore, the albumin and creatinine content, and the urinary volume of db/db mice were determined. As expected, both the ACR and the urinary volume were markedly increased in the db/db group. The LOE treatment significantly improved the ACR suggesting an improvement of the renal function in diabetic mice whereas no such effect was observed with the pioglitazone treatment.

Physical exercise is generally recommended for the treatment of T2DM because of its benefits on blood glucose control, insulin tissue sensitivity and weight management despite the fact that their exercise capacity is often impaired [61]. Therefore, the possibility that the LOE treatment might affect the physical exercise performance of db/db mice was determined using a treadmill. The exercise capacity of db/db mice, as assessed by the time to exhaustion and distance, was markedly lower than that of the db/+ mice, and both parameters were slightly but significantly increased by the LOE treatment but not by the pioglitazone treatment. The enhanced exercise capacity in the LOE-treated group most likely results from an improved metabolic state with a reduced body weight, improved blood glucose control and dyslipidemia, and an improved endothelial dysfunction and, hence, blood flow.

Altogether, the present findings indicate that LOE has a beneficial effect on T2DM targeting not only metabolic parameters such as hyperglycemia and dyslipidemia but also the endothelial dysfunction and vascular oxidative stress most likely by normalizing the angiotensin system. They further suggest that LOE might be of interest to prevent the development of micro and macrovascular complications in T2DM by improving the endothelial control of vascular tone and, hence, blood flow.

## Materials and Methods

### Ethics Statement

This study conforms to the Guide of Care and the Use of laboratory Animals published by the US National Institutes of Health (NIH publication No. 85–23, revised 1996) and the present protocol was approved by the local ethics committee (Comité Régional d’Ethique en Matière d’Expérimentation Animale, approval AL/01/09/09/05).

### Plant Extract and Standardization

The preparation of the extract of *L. obtusiloba* was prepared and standardized as described previously [27]. The dried stems of *L. obtusiloba* were collected in the vicinity of Hongcheon and were bought from Yakcho119 (Hongcheon, Republic of Korea). The voucher specimen (no. YJP-14) was deposited at the Herbarium of Korea Institute of Science and Technology (KIST, Gangneung, Kangwon-Do, Republic of Korea). *L. obtusiloba* was identified by Dr. Sang Hoon Jung (KIST, Gangneung, Kangwon-Do, Republic of Korea). Dried small branches were cut into small pieces and grounded into powder. The *L. obtusiloba* powder (15.8 kg) was extracted 4 times with 111 l of 50% aqueous ethanol (v/v) for 4 h at 70°C. The resultant was then evaporated to dryness under vacuum to produce 1.1 kg of a brown powder (yield of 7.0%). The concentration of polyphenols in the LOE extract was determined by the Folin-Ciocalteau assay and amounted to approximately 23.7% expressed as (−)-epicatechin equivalents. For HPLC analysis, 10 µl of LOE (10 mg/ml solubilized in 50% aqueous methanol) were injected into an Agilent 1200 series HPLC system. Separation was carried out using a 4.6×250-mm C18 reverse-phase column (Shiseido) maintained at 35°C and eluted at a flow rate of 1.0 ml/min using a mobile phase of 17% aqueous acetonitrile acidified with 0.1% trifluoroacetic acid. Chromatographic profiles were recorded at 254 nm. In order to identify the chemical structure of each peak, the ethanolic extract of *L. obtusiloba* was subjected to preparative RP-HPLC using a 250×20-mm i.d. 4-µm HiQSil column (KYA TECH) eluted with 17% aqueous acetonitrile to provide five major flavonoids. The chemical structure of the flavonoid compounds was elucidated by spectral analysis and direct comparison with authentic compounds. The identified compounds included hyperin, isoquercitrin, guaijaverin, avicularin, and quercitrin [27].

### Animal Treatments

For the present study, 14 male C57BL/KsJ Rj-db (db/+ mice, 5 week-old, 19–20 g) and 42 male C57BL/KsJ@Rj-db (db/db mice, 5 week-old, 20–22 g) were purchased from Elevage Janvier (Le Genest-Saint-Isle, France). db/+ mice were assigned into one group, and db/db mice were randomly assigned into three groups of 14 mice. Mice were housed in colony cages under standard laboratory conditions (12∶12 h light/dark cycle) and had free access to standard commercial diet and water. From week 6 until week 14, the groups of db/db mice received by daily gavage either vehicle (db/db group, 0.5% carboxymethylcellulose in 0.9% NaCl solution), LOE (db/db+LOE group, 100 mg/kg/day), or pioglitazone (db/db+Pio group, 30 mg/kg/day, DAEBONG LS., Ltd., Korea). The control group included 14 db/+ mice, which received the vehicle. LOE at a dose of 100 mg/kg/day has previously been shown to prevent angiotensin II-induced hypertension and endothelial dysfunction in rats [27], and pioglitazone at 30 mg/kg/day to preserve ß-cell mass in db/db mice [62].

### Measurement of Metabolic Parameters

From week 6 until week 14, non-fasting and fasting blood glucose levels were determined once a week using the Accu-Chek® Go (Roche Diagnostics, Indianapolis, IN, USA). Fasting blood glucose levels were determined 4 h after removing food. Food intake and body weight were also measured weekly. At week 14, mice were sacrificed, and organs were excised and their weight determined. Blood was collected directly from the heart in the presence of the anti-coagulant sodium heparinate and, thereafter, plasma was prepared and stored in aliquots at −70°C before being tested. Glutamic-oxaloacetic transaminase (GOT), glutamic-pyruvic transaminase (GPT), triglyceride (TG), total cholesterol (CHO), high-density lipoprotein (HDL), low-density lipoprotein (LDL) levels in plasma were determined using the clinical biochemistry analyzer Hitachi 7020 (Hitachi, Japan).

### Measurement of Urine Parameters

One week before sacrifice, mice were placed in metabolic cages for 24 h and thereafter, urine was collected for a 24-h period. Thereafter, urine samples were stored at −70°C before being analyzed. The albumin-creatinine ratio (ACR) in urine was measured by ELISA kits (Ref 50200, CellTrend, Germany).

### Vascular Reactivity Studies

The aorta was cleaned of connective tissue and cut into rings (2–3 mm in length). Rings were suspended in organ baths containing oxygenated (95% O_2_, 5% CO_2_) Krebs bicarbonate solution (in mM: NaCl 119, KCl 4.7, KH_2_PO_4_ 1.18, MgSO_4_ 1.18, CaCl_2_ 1.25, NaHCO_3_ 25, and D-glucose 11, pH 7.4, 37°C) for the determination of changes in isometric tension as described previously [27]. Briefly, rings were stretched step by step until the optimal resting tension of 1 g was reached and, then, were allowed to equilibrate for at least 60 min. After the equilibration period, the rings were exposed to Krebs bicarbonate solution containing high concentration of potassium (80 mM) until reproducible contractile responses were obtained. Thereafter, rings were contracted with phenylephrine (PE, 1 µM) to approximately 80% of the maximal contraction by the high potassium solution. After washout and a further 30-min equilibration period, rings were again contracted with PE before the construction of a concentration-relaxation curve to either acetylcholine (Ach) or sodium nitroprusside (SNP, an exogenous NO donor). In some experiments, rings were exposed to an inhibitor for 30 min before being contracted with PE. Relaxations were expressed as the percentage of the reversal of the contraction induced by PE. For the construction of the concentration-contraction curve to Ach, rings with endothelium were exposed to N^w^-nitro-L-arginine (L-NA, 100 µM) for 30 min and then contracted to approximately 10% with PE (100 nM).

### Determination of Vascular Reactive Oxygen Species Formation

The oxidative fluorescent dye dihydroethidium (DHE, Invitrogen Corp., Carlsbad, CA, USA) was used to evaluate the *in situ* formation of reactive oxygen species (ROS) using the method described by Miller et al. [63]. Aortic rings (3–4 mm length) from mice were embedded in OCT compound (Tissue Tek, Sakura, Torrance, CA, USA) and frozen in a liquid nitrogen bath. These unfixed frozen aortic rings were cut into sections (5 µm thick) and placed on polylysine-coated plus glass slides. After defrosting at room temperature for 60 min in a humidified chamber, DHE (2 µM in PBS) was applied to each section, which was then incubated in a light-protected humidified chamber at 37°C for 30 min before being mounted in Dako fluorescent mounting medium (Dako North America Inc., CA, USA). Sections were kept in the dark until fluorescence was determined using a confocal microscope (LSM510 META Carl Zeiss Inc., Overkochen, Germany). Quantification of the fluorescence intensity was performed using ImageJ software (NIH, USA).

### Immunofluorescent Staining

Frozen aortic rings embedded in OCT compound were cryosectioned at 20 µm and fixed with 4% paraformaldehyde. Fixed sections were incubated with antibodies directed against either eNOS (1/100, BD Transduction Laboratories, San Jose, CA, USA), NADPH oxidase subunits (p47phox and NOX-1, 1/100, Santa Cruz Biotechnology, Santa Cruz, CA, USA), nitrotyrosine (1/100, Santa Cruz Biotechnology, Santa Cruz, CA, USA), cyclooxygenase-1 (COX-1, 1∶200, Cayman Chemical Co., Ann Arbor, MI, USA), cyclooxygenase-2 (COX-2, 1∶200, Cayman Chemical Co., Ann Arbor, MI, USA), Ang II (1∶400, Bachem, Bubendorf, Switzerland), or angiotensin type 1 receptor (AT1R, 1∶400, Peninsula Laboratories Inc., San Carlos, CA, USA) overnight at 4°C. Immunoglobulin G coupled to Alexa 488 or 633 (Invitrogen Corp., Carlsbad, CA, USA) was used as secondary antibody. The level of fluorescence in each section was examined using a confocal microscope (LSM700 Carl Zeiss Inc., Overkochen, Germany). Images were analyzed by ImageJ software (NIH, USA).

### Measurement of Cyclooxygenase Activity

To determine the ability of LOE to inhibit COX-1 and/or COX-2 activities, a COX inhibitor screening assay (kit 560131; Cayman Chemical) was used according to the manufacturer’s recommendation. The 96-well plate was read in an automated microplate reader (VersaMax Plus) at 405 nm and the results were calculated following the instructions.

### Measurement of ACE Activity

Plasma ACE activity was measured by the rate of hydrolysis of the synthetic tri-peptide substrate FAPGG to FAP using ACE assay kits (Cat. #TR85056, TR85101, TR85201, Thermo Fisher Scientific Inc., Middletown, VA, USA) as described previously with some minor modifications [64]. 10 µl of ACE control, calibrator or plasma was added to 100 µl of ACE reagent in a 96-well micro-titer plate on ice, in triplicate. The reagents were mixed, incubated at 37°C and absorbance at 340 nm was read at 1 min intervals. The average absorbance readings of ACE activity in control, calibrator and plasma samples over time were plotted as linear regression graphs and gradients of these linear regression graphs ΔA/ΔT were calculated. The ACE activity of plasma samples was obtained by comparison of ΔA/ΔT for each sample with standards.

*In vitro* ACE activity was also assayed as described above with some minor modifications. LOE was dissolved in HBSS. Thereafter, 10 µl LOE or saline solution was added to 90 µl of ACE reagent in a 96-well micro-titer plate on ice in a dose-dependent manner, and the plate was mixed and incubated for 20 min. 10 µl of ACE control serum or saline was added to each well. The reagents were mixed, incubated at 37°C and absorbance at 340 nm was read at 1 min intervals for 5 min on an ELISA micro-plate reader. All samples were assessed in triplicate. Data were calculated as described above.

### Determination of Physical Exercise Capacity

Two weeks before sacrifice, mice were trained for 5 min at 15 cm/sec once a day on the treadmill for three consecutive days. Thereafter, the physical exercise capacity was assessed as the time and distance to exhaustion. The condition for measurement was at start 15 cm/sec and, thereafter, the speed was increased by 5 cm/sec per 1 min with an intensity of 0.3 mA.

### Chemicals

All chemicals were purchased from Sigma Chemical Co. unless specified.

### Statistical Analysis

Statistical analyses were performed using Student’s *t* test for unpaired data to compare two treatments. In the case of pairwise between-group comparisons, statistical analysis was carried out by one-way analysis of variance (ANOVA) followed by the Tukey post-hoc test. Concentration–response curves were compared by two-way analysis of variance (ANOVA) followed by Bonferroni post-hoc test. When the data consisted of repeated observations at successive time points, repeated measures one-way ANOVA followed by Tukey post-hoc test was applied to investigate differences between groups. All statistical analyses were conducted using Sigmaplot software (version 11) (SAS Institute, Cary, NC, USA). All results are expressed as means ± SEM. Differences were considered significant when *P*<0.05.
